# Why, When, and How to Treat Dynamic Forehead Lines with Botulinum Toxin Type A

**DOI:** 10.3390/toxins17120603

**Published:** 2025-12-17

**Authors:** Carla de Sanctis Pecora, Martina Kerscher, Mariana Muniz, Ada Trindade de Almeida

**Affiliations:** 1 Dermatologie Private Practice, Avenida Dr. Cardoso de Melo, 1308, Suite 121, São Paulo 04548-004, Brazil; 2 Division of Cosmetic Science, Department of Biochemistry and Molecular Biology, University of Hamburg, 20148 Hamburg, Germany; 3 Mariana Muniz Dermatology Private Practice, São Paulo 01405-100, Brazil; 4 Dermatology Clinic, Hospital do Servidor Público Municipal de São Paulo, São Paulo 01532-000, Brazil

**Keywords:** frontalis muscle, botulinum toxin, forehead wrinkles, customized treatment, uni-directional, bi-directional

## Abstract

Recent advances in the understanding of facial anatomy have contributed significantly to the refinement of injection techniques for the treatment of dynamic forehead lines. A comprehensive assessment of eyebrow shape, position, and the aging process is essential, as the latter are closely linked to the functional balance between the frontalis muscle and the upper facial depressors. Optimal outcomes also depend on the accurate determination of dosage per injection point, injection depth, and strategic distribution of injection sites within the frontalis, which should be carefully considered and tailored to the individual’s anatomical characteristics and therapeutic goals—whether the aim is neuromodulation for muscle activity reduction or intradermal application for skin quality enhancement. A round table discussion session among three experienced international dermatology experts in aesthetic botulinum toxin type A was performed during a MERZ LATAM-sponsored medical education session. Recent insights in facial anatomy, including the precise location and distribution of motor endplates, as well as the direction of muscular force vectors during contraction; aging processes; and interindividual variability in facial musculature and mimicry patterns are discussed, and the results are described herein. These factors play a critical role in customizing personalized injection strategies and improving aesthetic outcomes in the treatment of forehead lines.

## 1. Introduction

Cosmetic botulinum toxin A (BoNTA) injections are one of the most widely performed procedures in the aesthetic arena [1]. Initially intended to alleviate frowning lines, its indications rapidly expanded from the glabella to crow’s feet and to forehead lines in the upper face. The results helped injectors better understand the connection, interplay, and balance of the opposing forces of facial mimetic muscles [2].

The frontalis is an important mimetic muscle of the upper face. Its contraction elevates the eyebrows, shortening the forehead’s height and forming movements that translate emotions as surprise, fear, sadness, and attentiveness. Its unique positioning, without a bony attachment at its superior border, allows for dynamic interaction with the skin and subcutaneous tissues of the forehead.

Although frequently addressed in daily practice, the frontalis muscle and forehead region have gained attention in recent years through new anatomical [3], body painting and vector analyses [4] and ultrasonographic [5], biomechanical, and injection technique studies [6].

Rejuvenation of the forehead using neurotoxin injection is particularly challenging given the variations in anatomical, ethnic, and sexual dimorphism and functional features observed among subjects, in addition to different personal preferences and expectations related to the treatment, especially since this area has a prominent role in conveying emotion and expressing personality.

Thus, maintaining a natural facial look is of paramount importance. Careful assessment is required, taking into account the individual’s anatomy, variations in frontalis muscle mass and strength, eyebrow position and shape, and degree of peri-orbital laxity, particular movement habits, which may vary even in identical twins [7]. Achieving a balance among forehead wrinkle improvement, the upper face’s expression muscles, and the maintenance of the upper face’s structural characteristics—with special attention to the eyebrows’ shaping and positioning—is a key factor for successful outcomes. On the other hand, asymmetrical or dropped eyebrows can result in unpleasant and unsatisfactory results.

In this study, we review the related literature, discuss recent advances in the use of botulinum toxin in the forehead region, and contribute our experience and expert insights to help improve the neuromodulator treatment of this area. We discuss the following in this study as part of a round table on the use of botulinum toxin type A (BoNT-A) for the treatment of dynamic forehead lines in clinical practice: clinical anatomical pattern variations; total dosage and dosages per point; depth of injection; benefits of a customized treatment; and where to inject and when.

## 2. Results

### 2.1. Functional Anatomy

Female foreheads are straighter, usually with one or two eminences (round elevations). The supraorbital rim is less noticeable, and the glabella is less pronounced and curvier compared to males [8].

The upper facial muscles have cutaneous insertions and can be classified into two antagonistic groups: the eyebrow depressors, which comprise the *procerus*, *corrugator supercilii*, *depressor supercilii*, and *orbicularis oculi*; and the *frontalis*, the single eyebrow elevator [6]. The occipitofrontal muscle consists of two muscular bellies—the frontal and the occipital—connected and enveloped by dense connective tissue known as the epicranial aponeurosis (galea aponeurotica). The occipital belly pulls the scalp anteriorly, whereas the frontal belly elevates the eyebrows, leading to the formation of horizontal lines and interfering with the shape and position of the eyebrows. The *frontalis* is a large, thin, bi-belly, quadrilateral, and vertically oriented muscle of the forehead, with origins in the *galea aponeurotica* and insertions in the skin of the eyebrows and root of the nose, where it blends with brow depressors, such as the *procerus* and *orbicularis* [9]. The medial fibers of the frontalis bellies may be interlaced together for a variable distance above the root of the nose, while its lateral margin almost always ends or becomes markedly attenuated along or just lateral to the temporal fusion line [5]. The contraction of the *frontalis* raises the eyebrows, leading to the formation of horizontal lines and interfering with the shape and position of the eyebrows [9].

The correlation between the shape of forehead lines and the underlying anatomical pattern of the frontalis muscle has been demonstrated multiple times in the scientific literature. Based on anatomical findings and forehead line morphology, Moqadam et al. associated the “full-shape” frontalis pattern with straight, parallel lines, whereas the “V-shaped” pattern was linked to wavy horizontal forehead lines [10]. Abramo et al. compared the morphology of forehead lines in 20 living volunteers—both at rest and during active contraction—with forehead lines at rest and the anatomical pattern of the frontalis muscle in 20 cadavers [11]. The aim of this study was to establish a relationship between the cutaneous lines of the upper third of the face in cadavers and the skin lines produced by the voluntary contraction of the frontalis muscle in living subjects. This correlation was possible because the cutaneous lines in cadavers mirror the muscle dynamics that occurred during life. Four anatomical shapes have been described for the frontalis, namely, the full form, the V-shaped form, the central form, and the lateral form [11,12], which correlate with distinct clinical patterns of forehead wrinkles. The “full shape” pattern of the frontalis covers the entire forehead and is related to the appearance of straight parallel lines distributed over the entire region, whilst in the “V-shaped” frontalis—the muscle that is continuous inferiorly but separated in the mid-forehead—is linked to wavy horizontal forehead lines. In the “central” pattern, the aponeurotic tissue is located laterally, and the muscle is in the central part of the forehead, usually related to a column of lines in the central part of the forehead with the lateral part of the eyebrow dropped, while in the “lateral” shape, the two bands of the frontalis are completely separated with a large portion of aponeurotic tissue in the midline such that two columns of wrinkles placed laterally over the lateral ends of the eyebrows can be observed. Based on this knowledge, it is possible to interpret the appearance of the patient’s forehead lines and infer the shape, extension, and distribution of the frontalis muscle (FM) [6], thus guiding clinicians in determining optimal neurotoxin injection sites during forehead treatment.

### 2.2. Factors Influencing Outcome

#### 2.2.1. Why Should the Frontalis Be Treated with Neurotoxin Injection Apart from the Neuromuscular Indication?

Besides the intentional indication to improve forehead wrinkling, data suggest that toxin effects may extend beyond its intended short-term outcome on muscular activity. Mounting evidence demonstrates progressive, cumulative improvements in resting rhytids and skin quality after regular, repeated treatments with BoNT-A. Growing evidence suggests a cellular influence in the ongoing process of dermal repair, in addition to muscle relaxation [13], supporting the hypothesis that BoNT-A may have a direct or indirect effect on fibroblast activity [13] and the biomechanical properties of the skin, and it may enable dermal remodeling and improvements in elasticity, pliability, and radiance [14]. Nonetheless, it is important to point out that, to date, most data derive from low-evidence-level publications, and further randomized, double blind studies are still lacking. 

According to Humphrey et al., although clinical evidence for the use of BoNT-A for improvements of the skin may be limited, repeated treatment cycles with neurotoxin for facial wrinkles over a long period of time not only prevent new rhytid development but also improve skin quality and lead to the progressive reduction in established wrinkles [14]. Most available evidence derives from case studies. For instance, Binder et al. reported a 19-year follow-up of a pair of twin sisters. The sister who had been treated with BonT-A every 6 months during these 19 years presented much higher skin quality and no visible wrinkles at rest compared to the sister who had only been injected four times during the whole period [15]. Additionally, intradermal microdroplet techniques have been shown to improve skin texture, luminosity, pore size, and sebum production without significantly affecting frontalis function, likely due to the diffusion of the toxin into adnexal structures [16].

#### 2.2.2. Where to Inject?

The process of wrinkle formation and the biomechanics of facial muscles and their interaction with the overlying skin have long been areas of interest in both medical and aesthetic fields. Among the facial muscles, the frontalis muscle plays a crucial role in generating expressions, particularly through its involvement in eyebrow elevation and forehead wrinkling. As a primary muscle of facial expression, the frontalis is responsible for movements that convey emotions, such as surprise and attentiveness. Its unique positioning, without a bony attachment at its superior border, allows for dynamic interactions with the skin and the subcutaneous tissues of the forehead. The muscle fibers are directed superficially toward the skin, intermingling with the *orbicularis oculi* muscle.

The underlying muscle exhibits a specific degree of contractility, which is transmitted to the overlying skin. This transmission of mechanical forces between the muscle and the skin is mediated by a system of connective tissue fibers and fascial structures. In the forehead, this interface primarily comprises the supra-frontalis fascia and its associated subdermal fibrous architecture. Thus, skin displacement and the resulting formation of wrinkles represent the functional interplay between an intact connective tissue framework and underlying muscle contractility.

**Figure 1:**
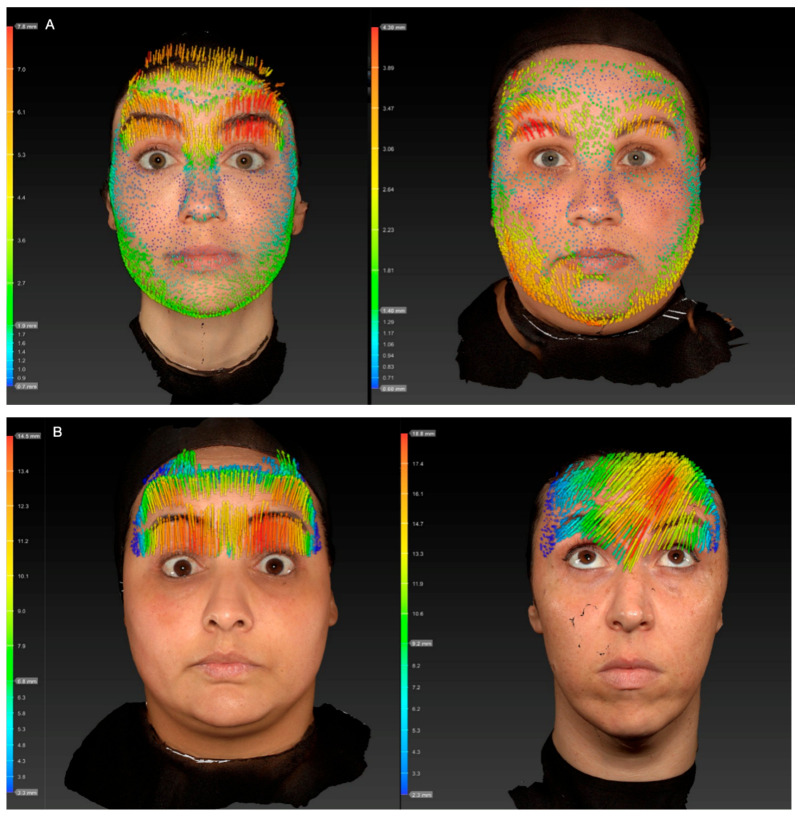
Processed three-dimensional scan (Vectra 1 camera system) representing the two different states (resting forehead, maximal contracted forehead) of the forehead, and their difference in skin displacement represented through colorful vectors. Inn the scale, red represents maximum displacement and blue minimum displacement. (**A**) Patients with bidirectional movement of the frontalis muscle. Observe the direction of the arrows downwards in the superior third of the forehead, whilst in the inferior two-thirds, arrows are directed upwards, representing a bidirectional movement (**B**) Patients with unidirectional movement of the frontalis muscle, where all the arrows are directed upwards, representing the displacement of the tissue towards the hairline.

Skeletal muscle contraction is unidirectional, occurs when the contractile force exceeds the opposing resistance, and follows force vectors that generally run from their insertion (mobile portion of the muscle) to their origin (fixed portion of the muscle), resulting in muscle shortening and the approximation of both ends. The latter mechanism results in hyperkinetic lines that are perpendicular to the direction of muscle contraction. This mechanical action reflects the sliding of thin filaments over thick filaments within the sarcomeres (actin and myosin), a process classically described by the sliding filament model of muscle contraction [17]. The action of the frontalis pulls the skin cranially, elevating the eyebrows and wrinkling the forehead in the process [18]. In 2018, Cohen and colleagues first suggested a bidirectional movement of the frontalis muscle when describing a non-surgical technique for forehead lifting (Figure 1) [19]. They postulated that as a vertically oriented muscle, it would shorten its fibers in an “accordion-like” effect, allowing displacements of the muscle fiber edges both at the origin and at the insertion. It was suggested in another publication that these two opposing movements of the frontalis muscle might converge to a line of convergence, named the C line [20]. Nevertheless, in our experience, this bidirectional movement can only be perceived in a small number of subjects. Another group demonstrated three distinct patterns of skin displacement during frontalis muscle contraction. In 16 participants (53.3%), only the lower portion of the forehead displaced upward. In 12 participants (40.0%), a bidirectional pattern was observed, characterized by the upward displacement of the lower forehead and downward displacement of the upper forehead. The transitional point for this bidirectional displacement occurred 59.4 mm above the pupil. In the remaining two participants (6.7%), the entire forehead demonstrated upward displacement during contraction [21]. Recently, a detailed anatomical study included clinical, ultrasonographic, and histological analyses; it found that the forehead may be divided into three zones or thirds. In the superior third, the FM is firmly attached to the deep galea, which, in turn, is not firmly adhered to the periosteum, allowing for minimum “passive” movement when pulled. In the middle third, no gliding plane exists, since the FM adheres to the deep galea, which is firmly attached to the periosteum. In the inferior third, the FM is separated from the deep galea by a fatty layer, and the galea is separated from the periosteum by areolar tissue [3]. The frontalis muscle movement occurs from the distal ends toward the mid-part of the muscle, contracting on its superficial layer and sliding over the deep part strongly attached to the deep fascia. In this study, they observed that the C line coincides with the union of the superior and the middle forehead zones [3]. Based on these recent findings, we question the existence of a bidirectional contraction of the frontalis muscle and raise the possibility of a bidirectional skin displacement secondary to the simultaneous contraction of the frontalis muscle and the occipital portion of the occipitofrontalis muscle. In this context, the two opposing movements would displace the forehead and scalp skin, respectively, potentially converging—in some patients—at the more adherent medial region. Further studies are needed to better understand this mechanism.

When treating a forehead with neuromodulators, it is important to remember that the skin of the brow has no bony attachment, and its position is determined by the balance of cranially oriented vectors of the frontalis muscle and caudally oriented vectors from the glabellar depressor complex, including procerus, *depressor supercilii*, *orbicularis oculi*, and *corrugator supercili* muscles [22].

A detailed assessment of the forehead’s dynamic wrinkles, muscle mass, and strength is vital to achieve a customized treatment of the forehead lines, creating a balance among the frontalis muscle fibers’ contractions [23]. The experts highlighted that the most important region of the FM for brow elevation is its inferior third. The other regions can also be addressed for wrinkling improvement and even forehead height extension, but they will not affect brow position. For this reason, the lower forehead must be treated carefully and precisely. It is well established that in order to minimize the risk of brow ptosis, injections in the forehead should not be placed within 2 cm of the orbital rim. When bidirectional frontalis activation is present, the convergence line is typically located at approximately 60% of the distance between the eyebrow and the hairline. Thus, provided that the 2 cm safety margin above the orbital rim is respected, botulinum toxin may be administered below the convergence line, enabling a more effective blockade of the frontalis muscle. In a retrospective study, 86 women were injected with incobotulinumtoxin A for the treatment of the glabellar and forehead wrinkles, including the lowest continuous horizontal frown line below the C-line. Considering muscle mass and strength distribution patterns and respecting the safety distance of 2 cm from the orbital rim, none of the participants had eyelid or eyebrow ptosis [24].

The experts agreed that botulinum toxin injection into the forehead should be individualized by considering the skin’s displacement and balance among the frontalis muscle and medial and lateral depressors. When the treatment of the upper portion of the inferior forehead is indicated, either to reduce lower forehead lines by limiting muscle activity or to modify eyebrow shape, neurotoxin injections may be safely administered using lower doses per injection point and a neurotoxin with a more precise diffusion halo [25], provided that the 2 cm distance from the orbital rim is maintained, irrespective of whether the patient demonstrates unidirectional or bidirectional frontalis movement. Furthermore, the assessment of the degree of brow and eyelid ptosis is also important when considering treating the lower part of the frontalis muscle.

#### 2.2.3. Depth of Injection

The depth of injection has also been the subject of controversy. It has been questioned whether the neurotoxin injection depth into the forehead would interfere with the effectiveness of the muscle contraction blocking effect. While some studies have deemed the more superficial injection technique safer than a deeper injection technique [26,27], in a prospective split-face study, the deeper injection yielded statistically significantly better outcomes [28]. In the latter study, BonT-A was randomly assigned to be injected in the superficial fatty layer (superficial to frontalis muscle) on one side, whereas on the contralateral side, the product with the same dose and number of injection points was delivered into the supra-periosteal plane (deep in the frontalis muscle).

Although the commonly injected volume of BoNT-A diffuses approximately 1 cm from each injection site (2 cm in diameter), in the superficial technique, BonT-A is separated by the intact fascia, which was not penetrated by the needle, limiting its efficacy to paralyze the muscle and explaining why the deep injection technique may result in a superior outcome.

The commonly injected volume of BoNT-A diffuses approximately 1 cm from each injection site (2 cm in diameter). A recent LATAM Consensus suggested intramuscular injections of tailored doses distributed in the entire frontalis according to muscle strength and recruitment [29]. Our advice on this matter is again to tailor the injection depth according to the patient’s needs. If partial paralysis is desired (e.g., actors, older patients with more laxity and thinner skin), superficial intradermal injections can be useful, lowering the risk of brow drop and heaviness sensation but at the price of reducing the duration of effects compared to intramuscular injections. For younger subjects or stronger frontalis muscles, intramuscular injections will be more effective and long-lasting.

#### 2.2.4. Dosage

On a molecular level, the efficacy of BonT-A relates to the amount of viable toxin available and to the percentage of neuromuscular junctions bound by active 150 kDa BoNT-A molecules. Inside the motor neuron, the N-terminal portion of the heavy chain inserts into the vesicle membrane, creating channels that allow the 50 kDa light chain to translocate toward the cytosolic side, where the latter is enzymatically released to bind to a member of the SNARE protein complex, specifically SNAP-25. The light chain exerts its lytic effects upon newly generated SNAP-25 throughout the light chain’s lengthy half-life of several months. Thus, the amount of active 50 kDa light chain domain in the motor neuron defines toxin longevity by cleaving SNAP-25 and allowing toxin to persist intracellularly in neuronal cells [30]. The more the receptors are bound by the active toxin, the stronger the clinical response will be. Both the short onset and long duration of effects are dependent on the concentration of toxin and the density of receptors. Thus, the quantity and density of receptors to which the neurotoxin binds directly impact efficacy, while patient factors, such as gender, muscle mass, facial structure, age, and genetics, affect the number of receptors [31]. The greater the muscle mass and strength, the greater the number and/or density of toxin receptors and the greater the amount of toxin required [30]. In summary, efficacy will vary based on the quantity of toxin injected, the treatment area, the degree of neuromuscular junction receptors available for binding, and ultimately, the desired cosmetic benefit to the patient.

Bearing in mind that effectiveness is mainly related to the amount of neurotoxin injected, it is important to consider not only the dosage per area but also the dosage per injection point.

Consensus guidelines propose dose ranges of 8–30 U for the treatment of dynamic forehead lines using onabotulinumtoxinA or incobotulinumtoxinA and 20–60 Speywood units for abobotulinumtoxinA. The dose allocated to each injection site may be individualized according to the frontalis muscle’s strength while remaining within consensus-recommended ranges to ensure therapeutic efficacy [31,32,33]. In clinical assessment, muscle strength can be estimated by palpation during active contraction and by evaluating wrinkle depth. In this consensus, dosing per injection point was classified as follows: 0.5 U for weak contraction, 1 U for moderate contraction, and 2 U for intense contraction. In male patients, doses may be increased by 1 U of incobotulinumtoxinA, respecting the average of total dosage suggested in the consensus.

Besides the traditional BonT-A use, “microtoxin” or “microbotox” injections have been described for the treatment of the forehead lines, especially in the lower frontalis, in an attempt to minimize adverse effects, such as eyebrow ptosis, by reducing the dosage per injection point, thus reducing the blocking effect [34]. “Microbotox” is characterized as multiple intradermal injections of microdroplets from 0.1 to 0.2 U/point with the objective of only blocking the superficial fibers of the frontalis muscle. Usually, a 100 U BoNT-A vial is diluted in 5 mL of saline solution for facial treatment [35]. The use of microdosages (dosage/point < 0.5 U) is particularly useful if the halo of spread is large—as with abobotulinumtoxin A compared with onabotulinumtoxin A and incobotulinumtoxin A—in order to increase safety while treating the inferior part of the forehead [25]. In a trial, eighty-six women had dynamic forehead lines injected with incobotulinumtoxin A, including the horizontal frown line below the C-line, with 0.5 U and 1 U of INCO per point in the inferior limit line without any eyebrow ptosis or eyelid ptosis being observed [24], reinforcing that a more precise halo of spread does not require the use of microdosages in the inferior limit such that the dosage per injection point is higher and more effective.

#### 2.2.5. Number of Injection Points

Injections targeted at the motor endplate seem to potentiate the effect of botulinum neurotoxin such that the determination of the motor endplate position and distribution would be crucial to improve the efficacy of neurotoxin injections. Concerning the upper facial musculature, studies on the distribution of motor end plates remain scarce despite their clinical relevance, particularly for therapeutic muscle weakening with botulinum neurotoxin (BoNT). Moreover, the interpatient variability needs to be considered. Existing evidence suggests that neuromuscular junctions in facial muscles are located near the entry points of the terminal branches of the facial nerve—distinct from the pattern observed in trunk and limb skeletal muscles. The literature mainly provides dissection-based descriptions of frontalis (FM) innervation, with limited detail on precise nerve entry points. Facial muscles typically show a cluster-like distribution of endplates in eccentric positions, except in the orbicularis oculi, orbicularis oris, and buccinator muscles. Neubert described the place and distribution of the motor end plates of the upper facial musculature by means of high-density surface electromyography. Frontalis muscle neuromuscular end plates are band-shaped and predominantly located in the middle and upper thirds of the muscle, arranged either uniformly in the mediolateral direction or divided into medial and lateral groups. In the medio-lateral direction, they were either evenly distributed in a band-shaped area or could be divided into medial and lateral clusters in one-third of the subjects. From most of the endplates, the propagation of activity in two opposite directions was detected. The superior fibers were oriented in a cranial and cranio-lateral direction; however, the propagation in the cranio-lateral direction increased with more lateral motor unit localization [36]. 

Additionally, the effectiveness of BoNT-A is greatest when the preparation is distributed in small aliquots throughout the muscle rather than as a single dose [37]. Thus, it has been postulated that a greater quantity of injection points can optimize toxin spread and distribution in a thin, broad, and flat muscle such as the frontalis, thereby resulting in a shorter onset and longer duration of effect compared with the injection of the same neurotoxin dosage divided into a smaller number of injection points [30]. Despite possibly increasing patient discomfort, increasing the number of injection points not only improves the distribution of the neurotoxin closer to the majority of motor endplates, allowing for full saturation of heavy chain receptors without “wasted” toxin [30] but also helps prevent the unnecessary distribution of toxin to tissues beyond the injection site. These data reinforced the need for a detailed assessment, the importance of spreading the injection points, and an individualized approach.

### 2.3. Especial Considerations

#### 2.3.1. Is There Any Situation Where We Should Not Treat the Frontalis with BonT-A?

While the benefit of BonT-A beyond its muscle relaxation properties has already been demonstrated, should all patients be treated for forehead wrinkles with BonT-A?

Over the past decade, there has been a progressive change in the concepts of aging and facial rejuvenation from an essentially two-dimensional focus to a three-dimensional approach that considers the volume of fat pads, bone structure, and collagen loss. Deep forehead wrinkles appear mainly as a consequence of the hyperkinetic muscles of the forehead, but other aging factors, such as the reduction in skin elasticity, flattening of the forehead, atrophy of fat pads, and eyelid ptosis, can contribute to the development/worsening of forehead wrinkles [6,38]. The more the frontal muscle is contracted, the shorter it becomes, and the deeper the forehead lines become. Eyelid and/or eyebrow ptosis may result in the development of horizontal forehead lines through compensatory frontalis activation [39].

In physiologic aging, there is a proportional compensation between the increase in frontal muscle activity and the antagonism of the depressor muscles (orbicularis, procerus, corrugator, and depressor supercilia muscles) such that the eyebrow position is unchanged [40]. For these subjects, neurotoxin injections for the treatment of the upper face’s wrinkles can be performed, keeping in mind that the balance between the frontalis and the medial and lateral depressors is key for a good outcome. However, if the eyebrow’s position has changed, this indicates that there is an imbalance between frontalis and depressor muscle activities. For instance, for eyebrow elevation caused by the overcompensation of the frontalis, BonT-A treatment can be performed with caution, recreating the balance among the levator and brow depressors, and consequently, a more natural appearance could be achieved. On the contrary, the presence of eyebrow ptosis indicates that, due to age-related changes, such as bony remodeling, fat pad atrophy, and tissue laxity, the compensatory action of the frontalis muscle has become insufficient. In such cases, neurotoxin injection into the frontalis is contraindicated.

#### 2.3.2. Eyebrow Asymmetry

A thorough assessment of eyebrow shape and position—both at rest and during maximal contraction—is essential before any neurotoxin-based treatment of forehead lines, and the achievable outcomes should be discussed with the patient in advance. Pre-existing eyebrow asymmetry may result from multiple factors, including differences in underlying bony structure, variations in eyebrow hair placement that alter perceived shape, and habitual muscle use resulting in unequal strength between the frontalis bands. Once the etiology is identified, the eyebrow’s position can be better balanced by modulating the functional relationship between the frontalis and the brow depressor muscles, primarily through the strategic distribution of injection points across the forehead and dose selection based on local muscle strength.

Palpation of the forehead during contraction is recommended; higher doses should be applied to areas displaying greater muscular activity.

When eyebrow asymmetry arises due to neurotoxin treatment for upper facial rhytids, we recommend reassessing eyebrow position during maximal frontalis contraction to identify asymmetric muscle activity requiring additional blockade, in addition to evaluating the maximal contraction of the orbicularis oculi—the lateral brow depressor. Administering 1–2 units of neurotoxin to the superior lateral fibers of the orbicularis oculi on the side of the depressed eyebrow may render brow elevation [41]. Flowchart S1 (see Supplementary Materials) provides a best-practice guide for aesthetic BoNT-A use when treating the forehead.

#### 2.3.3. Long-Term Use of BoNT-A: Consequences and Potential Benefits

One of the most frequent questions regarding repeated neurotoxin treatment for forehead lines concerns its long-term consequences and potential benefits. The available literature on this topic remains limited. Rzany et al. reported data from 4103 neurotoxin treatments administered to 945 patients who had undergone at least three consecutive treatment cycles [42]. Their study demonstrated no evidence of tachyphylaxis: treatment dose, injection intervals, and patient satisfaction remained stable throughout follow-up. Moreover, no cumulative adverse effects were observed; in fact, the incidence of adverse events decreased in later treatment cycles. Similarly, Carruthers et al. analyzed long-term data from 194 patients treated with onabotulinumtoxinA (ONA) for more than five years, totaling 5112 treatments with a mean follow-up of nine years [43]. The findings indicated that dosing remained relatively stable over time, injection intervals tended to increase, and the overall safety and efficacy profiles were consistently maintained.

### 2.4. Customization

With the increase in knowledge of the facial anatomy, as well as the available neurotoxins’ unique features, techniques that individualize treatment according to the patient’s needs and expectations are increasingly studied in aesthetic medicine.

**Figure 2:**
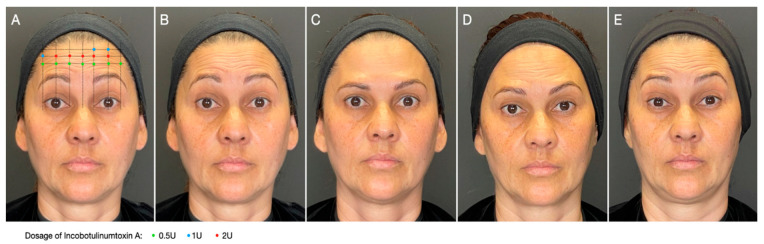
Customized approach for forehead wrinkles. Patient was treated with incobotulinumtoxin A using the ONE21 technique, distributing injection points and dosages over the contracting area of the frontalis according to the clinical anatomical pattern and strength distribution of the muscle. (**A**) Treatment scheme of the frontalis muscle using the ONE21 technique; (**B**) baseline picture: dynamic forehead lines, grade 3 on the Merz Aesthetic Scale (MAS); (**C**) 30 days after: 3-point improvement in MAS; (**D**) 120 days after: still 2-grade improvement in MAS; (**E**) 180 days after treatment: still showing some residual effect in brow position and shape.

The ONE21 technique (Figure 2) is a customizable injection protocol that allows for an individualized assessment and treatment of dynamic forehead wrinkles with BoNTA based on an understanding of the individual’s muscle anatomy and muscle contraction pattern, resulting in a predictable eyebrow shape (Figure 2) [6], which has been described in case series and three prospective open-label studies. A recent real-life case series described a customized treatment of the upper facial dynamic lines with incobotulinumtoxin A regarding the number and distribution of injection points, the total dosage, and the dosage per injection point. The forehead dynamic lines presented at least a 1-point improvement in 90%, 85%, and 70% of participants at D15, D90, and D120, respectively [44]. In an evaluator-blinded, open-label study, 28 women with facial movement lines underwent forehead wrinkle treatment with incobotulinumtoxin type A by two injectors following an individualized protocol (ONE21 technique) [45]. For dynamic forehead lines, 96%, 70%, and 67% of the subjects achieved a 1-point improvement in the MAS (Merz Aesthetic Scale) at day 30, 120, and 180, respectively.

The limitations of this study include the absence of formal methods, reliance on expert opinion, small or retrospective datasets, and potential sponsor influence.

## 3. Conclusions

Facial anatomy varies significantly between individuals in terms of structure, function, muscle mass, and strength—differences that are further influenced by gender, ethnicity, and age, resulting in a wide range of facial expressions. Consequently, standardizing injection points and doses may lead to suboptimal results, emphasizing the need for a more personalized approach. Understanding an individual’s frontalis anatomy and contraction pattern, muscle mass, and strength is essential for determining the most appropriate neuromodulator treatment for dynamic lines. Moreover, the customization of treatments, decisions on where to inject the frontalis muscle, choice of injection depth (intramuscular for dynamic wrinkle correction and intradermal for improving skin quality), and distributions of points and dosages should be carefully assessed to achieve the best possible esthetic outcome.

## 4. Methods

As part of the development of medical education during a MERZ LATAM-sponsored dermatology summit, a round table discussion session was conducted with 3 international dermatology experts with long-term experience in Aesthetic BoNT-A use, focusing on the treatment of forehead wrinkles with incobotulinum toxin type A, and a narrative summary of the results is described herein. No formal consensus was reached. The following topics were included in the discussion: recent advances in facial anatomy; the aging process; individual variations in facial musculature and mimicry and how these factors may impact the injection technique and aesthetic outcomes for patients with forehead lines; patient selection; detailed assessment; individualized treatment based on functional anatomy; injection technique; dosage; depth; number and distribution of injection points; and safety.

## Data Availability

The original contributions presented in this study are included in the article/Supplementary Material. Further inquiries can be directed to the corresponding author.

## Supplementary Materials

The following supporting information can be downloaded at https://www.mdpi.com/article/10.3390/toxins17120603/s1: Flowchart S1: Aesthetic guideline for BoNT-A application in forehead dynamic lines treatments.
