# Supplementary material for: Why, When, and How to Treat Dynamic Forehead Lines with Botulinum Toxin Type A
Source: Toxins (Basel). 2025 Dec 17;17(12):603. doi: 10.3390/toxins17120603 (PMC12737568; doi:10.3390/toxins17120603)
Supplement: Supplementary file 1 [file toxins-17-00603-s001.zip › toxins-3996015-supplementary.pdf]

Supplementary Materials: Why, When, and How to Treat Dynamic Forehead Lines with Botulinum Toxin Type A

Carla de Sanctis Pecora, Martina Kerscher, Mariana Muniz and Ada Trindade de Almeida

Flowchart S1. Aesthetic guideline for BoNT-A application in forehead dynamic lines treatments.

1. Baseline Assessment

1.1 Eyebrow Position

• Assess at rest and maximal contraction.
• Normal → proceed
• Asymmetry → manage (Section 1.2)
• Ptosis → contraindication for frontalis injection

1.2 Management of Eyebrow Asymmetry

Identify etiology:

• Skeletal asymmetry
• Eyebrow hair position / shape
• Muscular imbalance (frequent)

Correction strategy:

• Adjust dose asymmetrically.
• Modify depressor activity selectively.
• When toxin-induced:
◦ Evaluate frontalis contraction pattern and correct it injecting according to the contracting remaining area
◦ Add 1–2 U to the superior-lateral orbicularis oculi on the ptotic side.

2. Should the Frontalis Be Treated?

2.1 Determine Cause of Forehead Lines

• Hyperkinetic frontalis → Yes, treat
• Skin laxity, bone remodeling, fat atrophy → consider adjunctive treatments

2.2 Brow Ptosis Assessment

• If ptosis results from age-related structural changes, (bony remodeling, fat atrophy, tissue laxity, frontalis with weak contraction force unbalanced with brow depressors):
◦ Frontalis injection is contraindicated

3. Injection Zones

3.1 Safety Boundaries

• Never inject within 2 cm above the orbital rim

3.2 Contraction Pattern

• Unidirectional: Standard approach
• Bidirectional pattern:

◦ Identify convergence line (C-line ≈ 60% hairline–brow distance)
◦ Injection below C-line allowed if 2 cm safety margin maintained

3.3 Functional Zones of Frontalis

• Inferior third:
◦ Critical for brow position
◦ Inject conservatively
• Middle and upper third:
◦ Treat for rhytid improvement
◦ Minimal influence on brow position

4. Injection Depth

4.1 Choose Depth Based on Goal

• Superficial (intradermal/subdermal):
◦ Partial relaxation
◦ Useful for older patients, thin skin
◦ Lower risk of brow heaviness
• Deep intramuscular:
◦ Greater efficacy
◦ Longer duration
◦ Ideal for strong frontalis activity / younger patients

5. Dosage Strategy

5.1 Dose per Injection Point Based on Strength

• Weak contraction → 0.5 U
• Moderate → 1.0 U
• Strong → 2.0 U
• Male patients → consider +1 U per point

5.2 Total Dose Ranges

• OnabotulinumtoxinA / IncobotulinumtoxinA: 8–30 U
• AbobotulinumtoxinA: 20–60 Speywood U
• Adjust within consensus limits based on:
◦ Muscle mass
◦ Contraction pattern
◦ Desired cosmetic effect

6. Number of Injection Points

6.1 Rationale

• Frontalis = thin, broad, flat muscle
• Multiple small aliquots (0.5 - 1 - 2 - 3U) →
◦ Improved distribution
◦ Greater accuracy
◦ Better endplate coverage
◦ Longer duration
◦ Less undesired diffusion

6.2 Recommendations

- Use many small injections spaced across the muscle.
- Align point placement with areas of peak neuromuscular junction density (middle & upper thirds).

7. Special Techniques

7.1 Microbotox / Microdroplet Technique

- 0.1–0.2 U per point, intradermal
- Especially useful for:
  - High-spread products (ABO)
  - Inferior forehead where ptosis risk is higher

7.2 Product-Specific Considerations

- IncobotulinumtoxinA (INCO):
  - More precise diffusion halo
  - Microdosing unnecessary in lower forehead
  - Allows safer use of standard dosing per point

7.3 Customizable Techniques

- Example: ONE21 protocol
  - Maps individual contraction patterns
  - Balances frontalis vs. brow depressors
  - Predictable eyebrow shaping
  - Evidence shows high improvement in MAS scores at D30–D180

8. Long-Term Management

8.1 Repeated Treatments

- No tachyphylaxis demonstrated in long-term studies.
- Dose remain stable.
- treatment intervals remain stable or increase
- Incidence of adverse events decreases over time.

8.2 Benefits Beyond Muscle Relaxation

- Improvement in:
  - Resting rhytids
  - Elasticity
  - Dermal quality and radiance
  - Texture, pore size (microdroplets)
